# Wandering spleen with splenic torsion in a toddler: A case report and literature review

**DOI:** 10.1097/MD.0000000000022063

**Published:** 2020-09-11

**Authors:** Zhijun Wang, Qiang Zhao, Yuanyuan Huang, Zhanhao Mo, Zhisen Tian, Fan Yang, Yuanyi Wang, Liyu Yao

**Affiliations:** a Department of Pediatric Surgery; b Department of Pediatric Outpatient, The First Hospital of Jilin University; c Department of Radiology; d Department of Spine Surgery, China-Japan Union Hospital of Jilin University; e Department of Spine Surgery, The First Hospital of Jilin University, Changchun, China.

**Keywords:** pediatric surgery, splenectomy, splenic torsion, toddler, wandering spleen

## Abstract

**Rationale:**

Wandering spleen (WS) is a rare clinical entity characterized by splenic hypermobility caused by absent or abnormal laxity of the suspensory ligaments, which fix the spleen in its normal position. Due to abnormal attachment, the spleen is predisposed to torsion and a series of complications. Pediatric WS is mostly reported in children aged <10 years, especially among infants aged <1 year; it is uncommon among toddlers between 1 and 3 years. To the authors’ knowledge, only seven cases of WS have been described previously. Herein, we present the case of a 3-year-old toddler with WS and splenic torsion.

**Patient concerns:**

A 3-year-old boy was presented to the pediatric emergency room with a 2-day history of abdominal pain and vomiting. The ultrasonographic examination revealed a mass in the left upper abdomen cavity and absence of spleen in its normal position. Computed tomography showed an enlarged displaced spleen occupying the left abdomen cavity with an elongated splenic vascular pedicle (whirl sign), suggesting splenic torsion.

**Diagnoses:**

The patient was diagnosed that had WS and splenomegaly, with or without complications due to splenic torsion.

**Interventions:**

The patient underwent emergency laparotomy and splenectomy due to nonviability after detorsion.

**Outcomes:**

The postoperative course was uneventful, and the patient was discharged on the 7th day postoperatively without complications. The patient had favorable outcome over a 1-year follow-up.

**Lessons:**

Herein, we reported the case of a toddler with WS with splenic torsion. Moreover, after reviewing relevant studies in literature, we presented our findings on the diagnosis and treatment of toddlers with WS. Toddlers with WS are characterized by acute abdominal pain, unclear history description, examination restrictions, and high rates of life-threatening complications. High level of suspicion, careful physical examination, detailed history collection, and objective investigation are crucial in the management of toddlers with WS.

## 1 Introduction

Wandering spleen (WS) is a rare clinical entity characterized by splenic hypermobility caused by absent or abnormal laxity of the suspensory ligaments, which fix the spleen in its normal position.^[1]^ As the organ is only attached to an elongated vascular pedicle, the translocated spleen predisposes to torsion and serious complications. WS has an incidence rate of 0.2% and predominantly affects children aged <10 years and women in their third decade.^[2]^ In pediatric cases, the clinical presentation of WS usually includes acute abdomen and unspecific symptoms, such as nausea, vomiting, and fever. When WS is combined with splenic torsion, it may cause venous congestion and enlargement at the beginning. As the pathological course progresses, it may result in splenic infarction and ischemia; finally, splenic necrosis and rupture could occur.^[3]^ Pediatric patients can be divided into 7 subgroups according to their characteristics in different growing stages. Although children <10 years old are generally affected by WS, there are as many as 5 subgroups within the first decade. The term “toddlers” refers to children >1 and <3 years old, who begin to achieve more movement than infants, while keeping more immature characters than children in preschool years. Therefore, a review of toddler WS cases is necessary to investigate WS in pediatric patients.

Herein, we present a 3-year-old boy with WS with splenic torsion that was successfully treated by splenectomy. We have also reviewed the literature of all published toddler cases of WS to summarize essential information for the diagnosis and management of WS in toddlers.

## 2 Case report

Informed consent was provided to the patient's parents.

A 3-year-old boy was presented to the pediatric emergency room with a 2-day history of abdominal pain and vomiting. The pain was intermittent on the first day, and then turned to continuous and severe, before the patient was admitted to the hospital.

**Figure 1:**
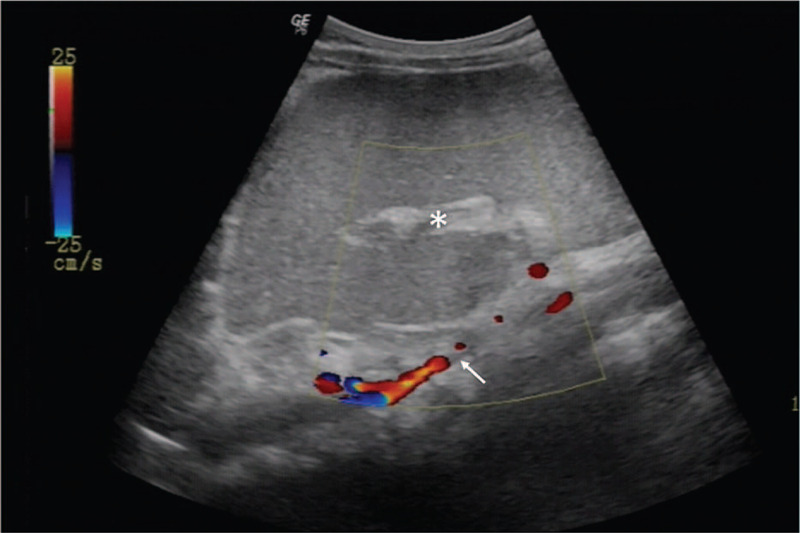
Ultrasound of the abdominal mass. The heterogeneous spleen presents a hyperechoic texture (marked in ^∗^) with absent intraparenchymal color Doppler flow from the twisting point on the vascular pedicle (arrow).

**Figure 2:**
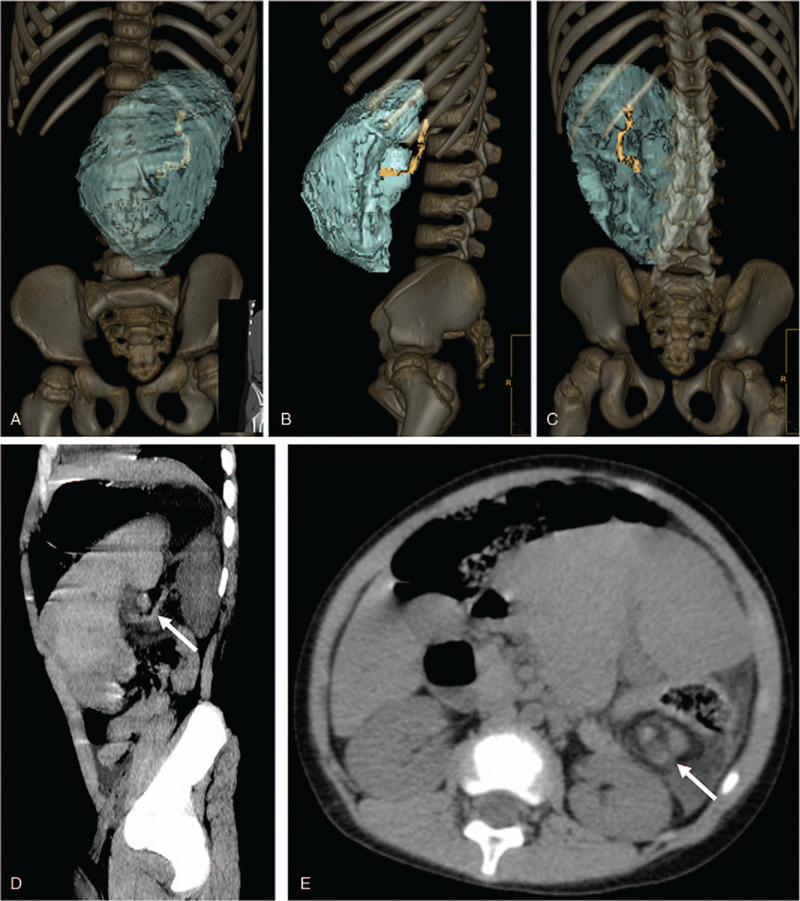
Abdominal CT with 3-dimensional reconstruction demonstrates an ectopic spleen, displaced in the left abdominal cavity. Abdominal CT with 3-dimensional reconstruction (A, B, and C) shows the spleen is enlarged, measuring 15 cm × 10 cm × 7 cm, the splenic vascular pedicle is elongated and flexed (colored in yellow). Sagittal plane of the abdominal CT shows that the splenic vascular pedicle is flexed (D, arrow). In the axial plane of the abdominal CT, the splenic pedicle presents a hyperdense and whirl appearance, which suggests that splenic torsion with surrounding fat accumulation is involved in the twisting (E, arrow). CT = computed tomography.

During physical examination, his body temperature was 37.6°C, heart rate was 144 beats/min, respiratory rate was 25 breaths/min, and blood pressure was 115/65 mmHg. Abdominal palpation indicated diffused tenderness, which was mainly found at the left abdomen to periumbilical region, with no rebound tenderness and guarding. Additionally, a firm, mobile mass with smooth surface was noted in the left upper abdomen, with the lower edge at about 10 cm from the lower margin of the costal arch, with obvious haphalgesia. An emergency ultrasonographic examination showed a mass in the left abdomen cavity and absence of spleen in its normal position. The splenic parenchyma was hyperechoic, with a significantly decreased color flow on Doppler (Fig. 1). Computed tomography (CT) with 3-dimensional reconstruction showed an enlarged displaced spleen occupying the left abdomen cavity, the distal end reaching the iliac crest (Fig. 2A–C). The CT scan also revealed a flexed elongated splenic vascular pedicle (Fig. 2A–E), which is shown as the whirl sign, with hyperdense parenchyma in the axial plane CT scan, suggesting the occurrence of splenic torsion (Fig. 2E). Further examinations including angiography and contrast-enhanced CT scan were rejected by the patient's parents to avoid the application of contrast agent. Hence, the patient was diagnosed with WS and splenomegaly, with or without complications due to splenic torsion.

**Figure 3:**
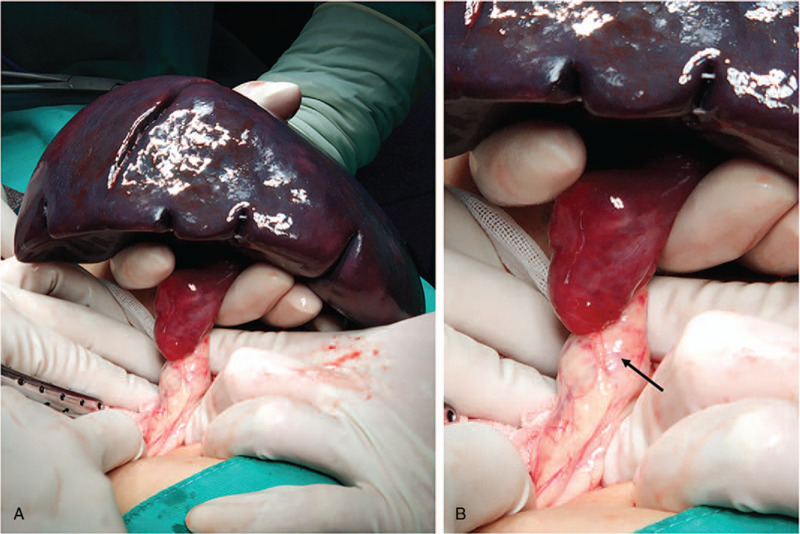
Resection of the wandering spleen. During the splenectomy, the spleen that revealed the absence of suspensory ligament was detected. The spleen was enlarged, measuring 15 cm × 10 cm × 6.5 cm, with complications of splenomegaly, congestion, and infarction (A). The splenic vascular pedicle twisted 720° and the proximal end of the pedicle involved the tail of the pancreas (B, arrow).

Emergency laparotomy was performed to ascertain the condition of the spleen. During the exploratory surgery, the absence of splenic suspensory ligaments was confirmed, and a 720°-twist vascular pedicle involving the tail of the pancreas in the torsion was identified (Fig. 3A and B). After manual detorsion, the tail of the pancreas was confirmed unharmed, whereas splenic infarction occurred without reperfusion. Therefore, splenectomy was performed due to nonviability.

The postoperative course was uneventful, and the patient was discharged on the 7th day postoperatively without complications. The patient had a favorable outcome over a 1-year follow-up.

## 3 Discussion

WS with torsion is a rare condition, accounting for <0.2% of all splenectomies.^[4]^ Although WS has been described from neonates to elderly patients in the 1980s, it has a 2-peak incidence that mainly occurs among children aged <10 years and women of reproductive ages (average: 25.2 years) among adults.^[5,6]^ In pediatrics, most WS cases are seen in children <1 year old, which is 2.5 times more common in boys than in girls,^[7]^ and the condition may have equal incidence rates in both sexes within the first 10 years of life.^[8]^ After the first decade, WS predominantly occurs among females compared to males, with a ratio of 7:1.^[9]^ However, within the younger occurring peak of WS, toddler (aged 1–3 years) WS cases are unusual. Among the known WS patients, only 8 toddler WS cases, including the present one, have been reported (Table 1). Among the reviewed cases, 4 patients were male and 3 were female (1 patient's gender was not reported). The average incidence age was 2.48 years; 6 cases’ incidence ages were above 2.5 years (30 months), which indicate a 1.5-year low-occurrence gap after the occurring peak in the 1st year.

**Table 1:**
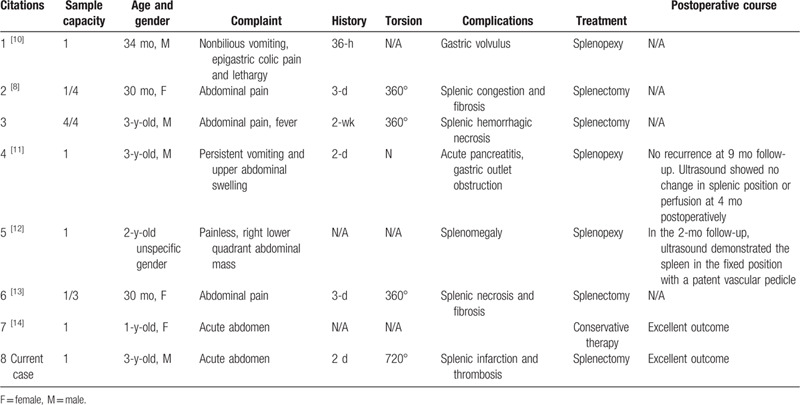
Review of toddler cases of wandering spleen.

The spleen is an organ that consists of vascular and lymphoid tissue. Normally, the spleen lies in the left hypochondrium of the abdominal cavity, while WS is defined as the spleen located outside its normal position.^[15]^ This condition is multifactorial, and the etiologies are broadly divided into congenital anomalies and acquired conditions.^[9]^ Generally, hypermobile spleen mainly results due to the absence or excessive laxity of suspensory ligaments; these ligaments include the gastrosplenic, splenorenal, splenocolic, splenophrenic, and pancreatico-splenic ligaments.^[9]^ Among these ligaments, the gastrosplenic and splenorenal ligaments attach the spleen to the stomach and posterior abdominal wall, respectively, and the splenocolic and splenophrenic ligaments support the spleen inferiorly, which are relatively critical in fixing the spleen in the splenic fossa.^[4]^ Congenital WS is mainly due to the failure of complete fusion of the mesogastrium and posterior abdominal wall during the second month of pregnancy, which may result in the absence or maldevelopment of one or more ligaments, and cause splenic hypermobility.^[1,15,16]^ Acquired forms occur secondarily to the diseases or conditions that may lead to laxity or damage of the suspensory ligaments. According to the reported cases, trauma, multiparity, connective tissue disorders, renal agenesis, and hormonal changes after pregnancy might increase splenic mobility.^[9]^ In pediatrics, congenital maldevelopment and abnormal fixation are the most common causes of WS.^[4]^ Acquired WS in pediatric patients is reported as being associated with multiple conditions, such as renal agenesis, immunoglobulin deficiency, infectious mononucleosis, malaria, Gaucher disease, Hodgkin lymphoma, and DiGeorge syndrome.^[3,4,17,18]^ In the published reviews of toddler WS cases, the patients were not reported with a WS-related history, suggesting that the younger the patient was, the more likely it was for them to have WS due to congenital anomalies than acquired conditions. In the current case, besides the twisted vascular pedicle, we have not seen any supportive tissue surrounding the wandering spleen, considering the patient without associate history with acquired forms, we suspected that he had congenital WS.

In a condition of an abnormal support of the ligaments, the spleen might be displaced from its normal position to anywhere in the abdomen or pelvic cavity. As a consequence of hypermobility, the splenic vascular pedicle, which is composed of the splenic artery and 6 more branches of the splenic vein, may be elongated and then twisted. The torqued splenic vessels can cause congestion within the entity, which leads to splenomegaly.^[19]^ In this condition, the pathological enlargement of the spleen may further worsen the hypermobility of the spleen and give rise to splenic torsion, which acts as a prime factor of splenic infarction. The elongated vascular pedicle and the hypermobile spleen predispose to torsion along the hilar axis, which is a life-threatening complication of WS in pediatrics. Depending on the torsion degree, it may subsequently lead to splenic infarction, gangrene, abscess formation, peritonitis, gastrointestinal obstruction, or spleen rupture.^[8]^ When the tail of the pancreas is partially twisted along with the vascular pedicle at the splenic hilum, it may cause pancreatitis and ischemia of the pancreatic tail. Among the toddler WS cases, 7 patients had different complications; 4 patients were diagnosed with WS combined with splenic torsion, and 2 patients’ spleen affected their stomachs (Table 1). Remarkably, although no torsion was identified, the elongated vascular pedicle was still able to encircle the gastric antrum, which resulted in acute gastric outlet obstruction and pancreatitis. In our case, the patient's vascular pedicle twisted 720°, which led to splenic infarction and thrombosis. Fortunately, although the pancreatic tail was involved in the torsion as well, it was not severely affected and was not damaged by the twisted splenic pedicle.

The clinical presentation of patients with WS varies from asymptomatic (with incidence diagnosis at one end) to an acute abdomen at the other end.^[20]^ Most adult patients with WS are asymptomatic and are incidentally diagnosed during physical examination, with a firm mobile abdominal mass with characteristic palpable “notched borders,” or via imaging examination for other purposes.^[9,21]^ Whereas pediatric patients usually present with acute abdominal pain, when complicated by splenic torsion, the pain severity often depends on the torsion degree. In pediatric patients, mild torsion may present as chronic abdominal pain due to splenic congestion; moderate torsion usually presents with intermittent pain, which is caused by torsion and detorsion of the vascular pedicle; severe torsion always leads to splenic infarction and results in acute abdomen.^[4]^ Among the toddler WS cases, 5 patients presented with acute abdominal pain, and when the pedicle encircled the stomach and obstructed the gastric outlet, the main complaint was persistent vomiting (Table 1). Due to their age, toddlers with WS experience difficulty in clarifying their symptoms; sometimes, “abdominal pain” with a palpable abdominal mass is the only clue for clinicians.

Compared to inquisition and physical examination, imaging examination such as abdominal ultrasound and computed tomography scan (CT) can provide more valuable information for diagnosis and preoperative evaluation of WS. In abdominal ultrasonography, during a condition of WS, the spleen is not visualized in the splenic fossa, and a mass appears in the abdomen or pelvic cavity, which demonstrates a heterogeneous or hypoechoic echotexture with decreased hilar color flow on Doppler.^[21]^ Beside the diagnosis of WS, the viability of the spleen, involvement of other nearby organs, and torsion degree, which can be defined by identification of the whirl sign and the hilar color Doppler flow, are able to be assessed via ultrasonography.^[6]^ Abdominal CT scan is another common examination that is routinely performed to aid the diagnosis and assessment of WS. Most common findings in CT scan include an empty splenic fossa and a translocated spleen.^[6]^ Contrast-enhanced CT angiography are helpful for a precise assessment of spleen perfusion, which is meaningful for surgical management. However, the toddlers with abdominal pain may have trouble in finishing a CT scan, let alone the invasive CT scans that require the injection of contrast material. In our case, the patient underwent abdominal ultrasonography and CT scan with the help of the parents, which demonstrated a heterogeneous mass in the left abdomen and a pedicle with the whirl sign, suggestive of torsion that might involve the tail of the pancreas and splenic infarction. However, as per the rejection of contrast agent, we failed to obtain more detailed information to evaluate the complications in spleen and other organs that were caused by WS, which challenged the planning of surgical strategy.

For patients who have mild symptoms and present no complications, conservative nonoperative management is the standard of care.^[22]^ However, it has been reported that more than 65% of patients with WS who underwent nonoperative treatment have developed complications such as splenic torsion, splenic injury, and injury of other organs, making conservative therapy nonadvisable in this condition.^[2]^ The choice of a surgical option is made according to the viability of the spleen; if the spleen shows reperfusion after manual detorsion, laparoscopic splenopxy may be offered, which is less invasive and can preserve the immunological function of the spleen and prevent postsplenectomy sepsis in children.^[6]^ Various laparoscopic spleenopxy techniques have been described in literature, and their common purpose is to reposition and fix the spleen to its normal anatomical position.^[23]^ Total splenectomy, partial resection via laparoscopy, or open surgery is recommended for patients with WS that have complications including splenic infarction, rupture, hemorrhage, thrombosis, splenomegaly, and compression of other organs.^[16,24]^ Postsplenectomy vaccinations against capsuled pathogens such as influenza and meningococcus are critical for pediatric patients. In the toddler WS cases, 7 of 8 patients were offered surgical treatment, among which, 3 underwent laparoscopic splenopexy, whereas 4 toddlers were offered splenectomy (Table 1). The high operating rate of toddlers with WS may attribute to self-ignorance among them; they and their parents might neglect the abnormal abdominal mass until acute abdomen occurs, which is a signal of worsened WS combined with complications. Therefore, medical common sense is important to young parents to help their children have early diagnosis and treatment of many conditions. The main limitation of our study is the lack of data due to the low incidence of WS among toddlers.

## 4 Conclusions

In summary, toddlers with WS are characterized by acute abdominal symptoms, unclear history description, examination restrictions, and higher rates of life-threatening complications. In our experience, high level of suspicion, careful physical examination, detailed history collection, and objective investigation are the basis for obtaining an accurate diagnosis and adapting appropriate treatment strategies of toddlers with WS, where preservation of the spleen is the primary goal.

## Author contributions

**Conceptualization:** Liyu Yao, Yuanyi Wang. Funding acquisition: Yuanyi Wang.

**Investigation:** Liyu Yao

**Project administration:** Yuanyi Wang.

**Resources:** Zhijun Wang, Qiang Zhao, Yuanyuan Huang.

**Software:** Zhanhao Mo, Zhisen Tian.

**Supervision:** Yuanyi Wang, Liyu Yao.

**Validation:** Fan Yang.

**Visualization:** Zhisen Tian, Yuanyuan Huang.

**Writing – original draft:** Zhijun Wang, Qiang Zhao, Yuanyi Wang.

**Writing – review & editing:** Yuanyi Wang.
